# 575. Local Experience of Breakthrough SARS-CoV-2 Infections After Full Vaccination

**DOI:** 10.1093/ofid/ofab466.773

**Published:** 2021-12-04

**Authors:** Ahmed A Khan, Gayla Havener, Donald Graham, Brian Miller, Gail O'Neill, Vidya Sundareshan

**Affiliations:** 1 Southern Illinois University School of Medicine, Springfield, Illinois; 2 Sangamon County Department of Public Health, Springfield, Illinois; 3 Sangamon County Department of Public Health/Springfield Clinic, Springfield, Illinois

## Abstract

**Background:**

SARS-CoV-2 the etiology of COVID-19 has caused more than 33 million cases and almost 600,000 deaths in the United States alone. Vaccination is a vital tool in controlling the pandemic. With accelerated infection rates in various parts of the world, the incidence of variants has risen and threatens to set back the long sought after immunity, provided by available vaccines. The objective of this study was to evaluate the breakthrough infection rate after complete vaccination, in Sangamon County, with a rural and urban population of 195,000 in Central Illinois.

**Methods:**

Data regarding breakthrough infections collected from the Sangamon County Department of Public Health, included the total number of infections, time after vaccination, age range of those infected and the type of vaccine used. Complete vaccination was defined as 14 days after the single dose of Johnson & Johnson/Janssen or the second dose of Pfizer-BioNTech or Moderna Inc. vaccine.

**Results:**

The number of fully vaccinated individuals at the time of writing of this study was 87,086 which corresponded to 44.58 % of the total population. The breakthrough infection percentage was calculated as 0.036%. The mean time after vaccination to infection was 49.13 days with a standard deviation of 23.28.

**Conclusion:**

Breakthrough infections among fully vaccinated individuals in our county, have been quite rare, which points to the high efficacy of the vaccines. A complex number of factors likely contribute to this including virus-related factors i.e. variant forms and specific patient-related factors which are not a part of this study. The afore-mentioned high efficacy rate of the vaccines provides further justification, to continue to pursue a persistent vaccination strategy to mitigate the effects of the SARS-CoV-2 virus.

**Disclosures:**

**All Authors**: No reported disclosures

**Table:** 

Vaccine	Infections	% of Breakthrough
Pfizer-BioNTech	14	43.75
Moderna, Inc.	10	31.25
Johnson & Johnson/Janssen	8	25
Total	32	100

